# Inflammatory Myofibroblastic Tumor of Sigmoid Colon: Unusual Cause of Intestinal Obstruction

**DOI:** 10.7759/cureus.11809

**Published:** 2020-11-30

**Authors:** Sakthivel Chinnakkulam Kandhasamy, Sudharsanan Sundaramurthi, Chellappa Vijayakumar, Mangala Goneppanavar, Vishnu Prasad Nelamangala Ramakrishnaiah

**Affiliations:** 1 Surgery, Jawaharlal Institute of Postgraduate Medical Education and Research, Puducherry, IND; 2 Pathology, Mahatma Gandhi Medical College and Research Institute, Puducherry, IND

**Keywords:** fibroblast, inflammatory myofibroblastic tumor, myofibrobalst, sigmoid colon

## Abstract

Inflammatory myofibroblastic tumors (IMFTs) are rare solid mesenchymal tumors frequently noted in children and young adults. It is characterized by variable clinicopathological and etiopathogenetic features. They are commonly reported in the lungs and occurrence in the colon is extremely rare. Here, we report a case of IMFT in the sigmoid colon confirmed histopathologically after surgical resection.

A 40-year-old lady presented with abdominal pain, vomiting, and constipation for four days. On abdominal examination, there was tenderness in the left iliac fossa region with localized guarding. Contrast-enhanced computed tomography (CECT) showed a sigmoid colonic mass lesion with few enlarged perilesional lymph nodes. Colonoscopy demonstrated circumferential ulceration with irregular margin associated with luminal narrowing noted 55 cm from the anal verge and scope could not negotiate beyond, biopsies were taken. Later, the biopsy came as descriptive in nature. Hence, we proceeded for surgery and intra-operatively we have found there was circumferential thickening in the sigmoid colon for about size 8 cm of which was abutting the left lateral parietal wall. We have done sigmoid colon resection with adequate margins and postoperatively patient did well. Finally, the histopathology report suggested an IMFT sigmoid colon.

## Introduction

Inflammatory myofibroblastic tumor (IMFT) is an unusual solid tumor of mesenchymal origin and was grouped into a mixture of fibroinflammatory disorders. It consists of fibroblasts and myofibroblasts, which are mixed with inflammatory cells, mostly of mononuclear type. IMFT is commonly reported in the lungs, and its occurrence at other extrapulmonary sites is rare. Uncertainty of origin has resulted in various synonyms consisting of inflammatory pseudotumor, plasma cell granuloma, plasma cell pseudotumor, pseudosarcomatous fibromyxoid lesion, pseudosarcomatous myofibroblastic lesion, and inflammatory myofibrohistiocytic proliferation. All the entities share common pathological features, including the variable spread of inflammatory cells with heterogeneous spindle cells [1]. Herein we report a rare case of IMFT of the sigmoid colon, which presented with colonic obstruction.

## Case presentation

**Figure 1:**
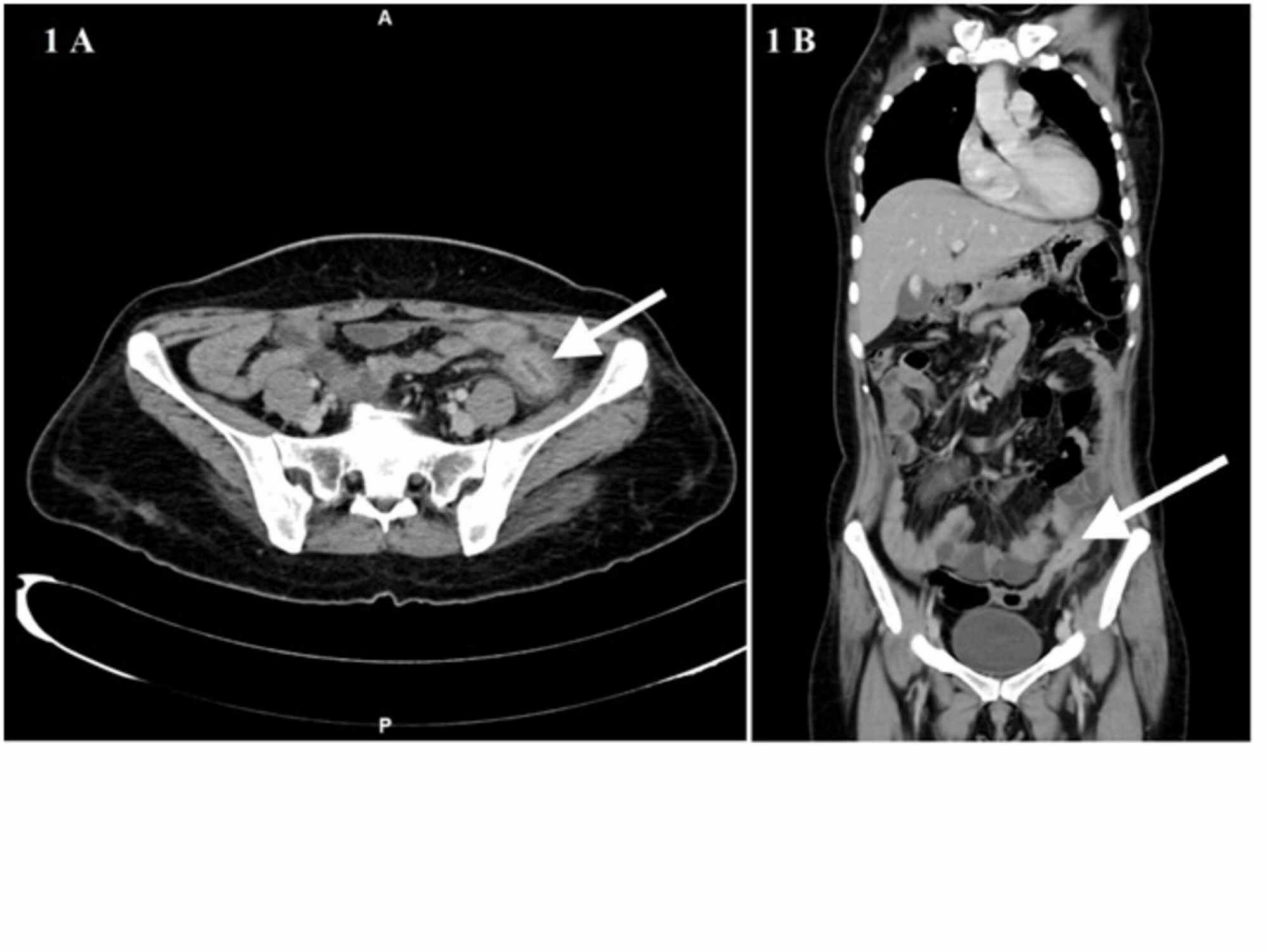
CECT of the abdomen axial plane (A) and coronal plane (B) show concentric thickening with mucosal hyperenhancement in the proximal sigmoid colon (white arrow) CECT: contrast-enhanced computed tomography

A 40-year-old lady presented to the surgical emergency with the complaints of abdominal pain, vomiting, and constipation of four days duration. She did not give a history of hematemesis and or melena. She had a history of a cerebrovascular accident with right hemiparesis and was on aspirin 150 mg for the past 10 years. On examination, her vitals were stable; per abdomen, there was tenderness in the left iliac fossa with localized guarding. Laboratory investigations suggested microcytic hypochromic anemia, elevated erythrocyte sedimentation rate (ESR), leukocytosis, and a normal platelet count. Contrast-enhanced computed tomography (CECT) showed concentric thickening with mucosal hyperenhancement noted in the proximal sigmoid colon for a length of 6 cm with a maximum thickness of 7 mm. There was surrounding fat stranding, and few enhancing pericolic lymph nodes were seen (Figure 1A and 1B).

**Figure 2:**
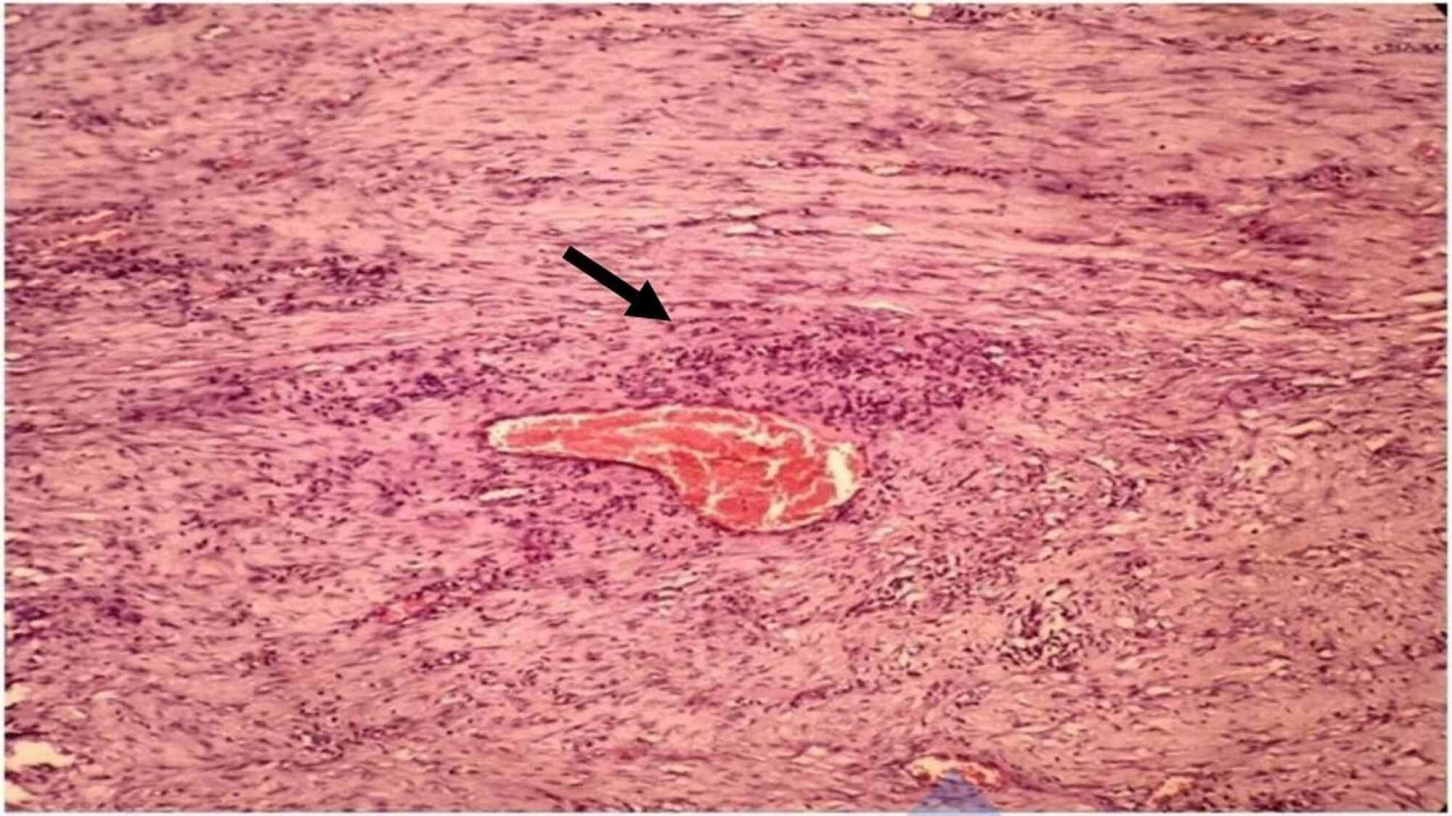
Histopathology shows perivascular hypocellular spindle cells intermixed with lymphocytic infiltrates (black arrow)

Colonoscopy showed a circumferential ulceroproliferative growth with luminal narrowing noted at 55 cm from the anal verge. Multiple biopsies were taken. The histopathology was inconclusive. The patient was planned for exploration, and on laparotomy, a malignant appearing growth was noted in the sigmoid colon for a length of 8 cm. There was no evidence of metastatic deposits. Sigmoid colon resection was done with adequate margins. Histopathology of the specimen revealed a colonic wall infiltrated with tumor arranged in loose fascicles composed of spindle cells displaying minimal atypia, fine chromatin, visible nucleoli, and a moderate amount of basophilic cytoplasm. The tumor cells were intimately admixed with plasma cells, lymphocytes, and eosinophils (Figure 2).

On immunohistochemistry, tumor cells were diffusely positive for smooth muscle actin and were negative for CD 34 and ALK1. Finally, it was confirmed as IMFT of the sigmoid colon. The postoperative period was uneventful. The patient was followed up for 18 months and is free from recurrence.

## Discussion

In 1937, IMFT was initially described in the respiratory system and had evolved from an inflammatory reaction to a lesion with intermediate malignant potential. The lungs are the most commonly affected sites, and various extrapulmonary presentations have been reported. The involvement of mesentery and omentum constitutes 43% of cases and holds for the most frequent extrapulmonary site [1]. Less than 30 cases of IMFT have been reported in the colon [2]. Children and young adults are the most commonly affected age group, but recently it has been documented in age extremes. The mean age group affected was ten years. There is no definite gender difference in the incidence of cases [1]. 

The etiology of IMFT is poorly understood. Various factors have been implicated in the development of IMFT, such as surgery, trauma, autoimmune reaction, infection, especially Epstein-Barr virus, and human herpes virus. It frequently presents with locally aggressive behavior and is ideally considered a neoplastic process. DNA aneuploidy, interleukin-6 overexpression, and cyclin D1 expression have been noted in some cases. Cytogenetic alteration in the ALK1 gene located at chromosome 2p23 has been linked to these tumors' development in 50% of the cases. Constitutional activation of the ALK1 gene encodes tyrosine kinase receptors and has potential oncogenic activity [3]. Despite negative ALK1 status, our case has been free from recurrence in the follow-up period.

Colonic IMFT manifests depending on their sites of occurrence. Colonic IMFT, albeit a rare entity, often affects the right colon. Contrastingly, in our case, it has occurred in the sigmoid colon. Elevated erythrocyte sedimentation rate (ESR), leukocytosis, thrombocytosis, and hypergammaglobulinemia are the laboratory findings that may be noted in some cases [4]. In our case, ESR and total leukocyte count returned to baseline after three weeks of surgical removal of the tumor. Although nonspecific, these may reconcile after surgery and can be used as an untimely indicator of tumor recurrence.

IMFT shares similar pathological findings of the desmoid tumor, leiomyoma, leiomyosarcoma, inflammatory fibroid polyp, gastrointestinal tumor, and schwannoma [5]. Inflammatory fibroid polyps are usually found in the sub-mucosal locations and constitute spindle cells, granulation tissue, and eosinophils. Fibromatosis consists of spindle and or stellate cells present in the collagen background and often have mitotic activity [6]. Gastrointestinal stromal tumor characteristically stains positive for CD117 and CD34 [7]. In contrast, leiomyoma and leiomyosarcoma stain positively for actin and desmin. Schwannoma stains strongly for S-100 and negative for CD100. Even though it is not specific, monoclonal antibody detection against ALK1 protein is identified in 50% of cases. Several studies described that ALK1 positive tumors are less aggressive, and no clear relationship with the prognosis can be made [1].

Multiple treatment modalities have been described for IMFT, but surgery is the treatment of choice. Complete surgical excision with an adequate negative margin has less than 10% chances of recurrence [3]. At present, there is no evidence to support chemoradiation for routine use. It may be reserved for inoperable tumors, post incomplete resection, and in cases with positive margins after resection. Chemotherapy, combined with oral non-steroidal anti-inflammatory drugs, maybe a therapeutic option in unresectable tumors. In our case, less aggression of the disease may be due to the long-term administration of aspirin therapy for stroke, which may be an added therapeutic advantage. Corticosteroids can be added to reduce the inflammatory reaction. Extrapulmonary location, especially in the abdomen, locally invasive lesions, and more than 8 cm tumor size, often has a high propensity for recurrence [8]. The recurrence rate is meager and regular follow-up is necessary for the long term to avoid it.

## Conclusions

Colonic IMFTs are solid mesenchymal tumors with intermediate malignant potential. With very limited prognosticators, surgery with a negative margin is the standard of care in the present scenario. It is not clear regarding the efficient treatment for local recurrence and metastasis and necessitates a long-time follow-up even after adequate negative surgical margins. Serial radiological and ESR estimation may help assess early recurrence.
